# Transgressions Are Equal, and Right Actions Are Equal: some Philosophical Reflections on Paradox III in Cicero’s *Paradoxa Stoicorum*

**DOI:** 10.1007/s11406-016-9761-4

**Published:** 2016-10-05

**Authors:** Daniel Rönnedal

**Affiliations:** Department of Philosophy, Stockholm University, Stockholm, Sweden

**Keywords:** Stoic paradoxes, Stoicism, Cicero, Dyadic deontic logic

## Abstract

In *Paradoxa Stoicorum*, the Roman philosopher Cicero defends six important Stoic theses. Since these theses seem counterintuitive, and it is not likely that the average person would agree with them, they were generally called “paradoxes”. According to the third paradox, (P3), (all) transgressions (wrong actions) are equal and (all) right actions are equal. According to one interpretation of this principle, which I will call (P3′), it means that if it is forbidden that A and it is forbidden that B, then not-A is as good as not-B; and if it is permitted that A and it is permitted that B, then A is as good as B. In this paper, I show how it is possible to prove this thesis in dyadic deontic logic. I also try to defend (P3′) against some philosophical counterarguments. Furthermore, I address the claim that (P3′) is not a correct interpretation of Cicero’s third paradox and the assertion that it does not matter whether (P3′) is true or not. I argue that it does matter whether (P3′) is true or not, but acknowledge that (P3′) is perhaps a slightly different principle than Cicero’s thesis. The upshot is that (P3′) seems to be a plausible principle, and that at least one part of paradox III in Cicero’s *Paradoxa Stoicorum* appears to be defensible.

## Introduction

In *Paradoxa Stoicorum* the Roman philosopher Marcus Tullius Cicero defends six theses that appear to have been an important part of Stoic philosophy in antiquity (Cicero (1874, 1942, 2004), Webb (1985)).^1^ Since these theses are prima facie counterintuitive, and it is not likely that the average person would agree with them, they were generally called “paradoxes”. This paper concerns the third paradox:

(P3) Transgressions are equal, and right actions are equal.^2^

What exactly did the Stoics mean by this principle, and how did they defend it? Did Cicero himself believe in (P3)?^3^ I will not try to answer these historical questions in this paper. Instead, I will focus on the philosophical plausibility of the principle. I will consider one interesting interpretation of (P3), which I will call (P3′), and I will show how (P3′) can be proven in dyadic deontic logic. According to (P3′), if it is forbidden that A and it is forbidden that B, then not-A is as good as not-B; and if it is permitted that A and it is permitted that B, then A is as good as B. I also try to defend (P3′) against some philosophical counterarguments. Furthermore, I address the claim that (P3′) is not a correct interpretation of Cicero’s third paradox, and the assertion that it does not matter whether (P3′) is true or not. I argue that it does matter whether (P3′) is true or not, but acknowledge that (P3′) is perhaps a slightly different principle than Cicero’s thesis. The upshot is that (P3′) seems to be a plausible principle and that at least one part of paradox III in Cicero’s *Paradoxa Stoicorum* appears to be defensible.

There are several good reasons to be interested in (P3):

1. The thesis is part of one major historically influential philosophical school, namely Stoicism. Accordingly, the topic of this paper is historically significant.
2. The principle is philosophically interesting in itself, regardless of its place in Stoic ethics. On the face of it, the thesis is implausible. However, on closer examination it is neither obviously true nor obviously false; hence, it merits closer investigation. Our discussion will suggest that the first part of (P3) might be false, but the second part true, and that both parts of (P3′) are true. Furthermore, if the thesis is true it seems to have genuine consequences for how we ought to live.
3. We also have good logical reasons to be interested in the thesis. Since a particular version of (P3), namely (P3′), is provable in dyadic deontic logic, it can be used to test the philosophical plausibility of certain deontic systems. If there are instances of (P3′) that are false, it seems that we must either reject the definitions of “better than” and “equally good” used in this paper, or abandon the kinds of dyadic deontic logic that are employed in our proofs.
4. As far as I know, there are no serious philosophical discussions about (P3) in the literature. The proofs of our particular interpretation of (P3), namely (P3′), are entirely new. The problems with (P3′) (and (P3)), that are discussed in this paper, are similar to some arguments mentioned already by Cicero. However, I believe that the counterarguments introduced in Section 3 are stronger in at least one interesting sense, and therefore worth closer scrutiny.

In conclusion, I think that the current investigation is well motivated.

To prove (P3′), I will use dyadic deontic logic. Deontic logic is a branch of logic that deals with normative words, such as “ought”, “right” and “wrong”, normative sentences, arguments and systems. Introductions to this branch can be found in, for example, Gabbay, Horty, Parent, van der Meyden & van der Torre (eds.) (2013), Hilpinen (1971, 1981), and Åqvist (1984, 1987, 2002). Dyadic deontic logic is a kind of deontic logic that includes several dyadic operators that can be used to symbolise various conditional norms. Rescher (1958), von Wright (1964), Danielsson (1968), Hansson (1969), van Fraassen (1972, 1973), Lewis (1973, 1974), von Kutschera (1974) and Åqvist (1971, 1973, 1987) are some of the pioneers in this branch of logic. In systems of this kind, several value operators can be defined in terms of the primitive dyadic deontic operators. The different value operators can then be utilized to symbolise such important value expressions as “is better than”, “is at least as good as” and “is as good as”. In the present paper, I will employ a dyadic deontic system, introduced by Rönnedal (2009), to prove (P3′). This system is called “TG”. I use TG because it is sound with respect to an intuitively plausible semantics. However, it is also possible to prove (P3′) using several weaker systems.

The paper is divided into four sections. Section 2 includes some reflections on how (P3) will be interpreted in the present paper, and a proof of (P3′). According to our reading of (P3), (P3′), this thesis contains two parts: (i) If it is forbidden that A and it is forbidden that B, then not-A is as good as not-B; and (ii) if it is permitted that A and it is permitted that B, then A is as good as B. In Section 3, I discuss some possible philosophical counterarguments to this principle. I consider two arguments: one against part (i) of (P3′) and one against part (ii) of (P3′), and I try to respond to these counterarguments. In Section 4, I address the claim that (P3′) is not a correct interpretation of Cicero’s third paradox, and the assertion that it does not matter whether (P3′) is true or not. I try to show that (P3′) has important practical consequences, and that it does, or should, matter to us whether it is true or not. I acknowledge that there might be other interpretations of the first part of (P3) that are more in tune with Cicero’s own views. This, however, does not imply that (P3′) is not an interesting principle. Furthermore, it is difficult to know what the Stoics themselves thought. Finally, Section 5 contains a short summary of the paper. I conclude that (P3′) seems to be a plausible principle. Furthermore, there appear to be good reasons to accept at least the second part of paradox III in Cicero’s *Paradoxa Stoicorum*.

## Proof of Paradox III in Cicero’s *Paradoxa Stoicorum*

According to (P3), (all) transgressions (wrong actions) are equal and (all) right actions are equal. There are many possible interpretations of this thesis. According to one interesting reading, which is the focus of this paper, it means the following:

(P3′)

(i) If it is forbidden that A and it is forbidden that B, then not-A is as good as not-B.
(ii) If it is permitted that A and it is permitted that B, then A is as good as B.

Note that part (i) in (P3′) is not equivalent to the following principle (i′): If it is forbidden that A and it is forbidden that B, then A is as bad as B. If we replace (i) by (i′) in (P3′), we obtain a different interpretation of (P3) that I will call (P3′′) (see Section 4.1). (P3′′) is perhaps a better interpretation of the Stoic principle. However, I will focus on (P3′), since we can prove part (i) in the system TG, but we cannot prove (i′). Both (i) and (i′) are prima facie counterintuitive, and both principles seem to be equally interesting. In Section 4.1, I return to the question of whether (P3′) or (P3′′) is a better interpretation of (P3). If we replace “forbidden” with “wrong” and “permitted” with “right” in (P3′), I believe we obtain an equivalent principle. So, I use “permitted” and “right” as synonyms in this paper. I think that this is the standard view among deontic logicians and moral philosophers.^4^ Since A is as good as B if and only if (iff) B is as good as A, we can also say that A and B are equally good if A is as good as B (i.e. they are “equal”), and likewise for not-A and not-B. “A” and “B” in (P3′) can stand for any sentences, including various action sentences. Replace “A” with “You kill your brother” and “B” with “You kill your sister”. Then the following is an instance of (P3′) (i): If it is forbidden that you kill your brother and it is forbidden that you kill your sister, then not killing your brother is as good as not killing your sister. Let A stand for “You help your brother” and B for “You help your sister”. Then the following is an instance of (P3′) (ii): If it is permitted that you help your brother and it is permitted that you help your sister, then helping your brother is as good as helping your sister.

To be able to prove (P3′), we will have to know a little bit more about dyadic deontic logic. The system TG includes all classical truth-functional connectives, three modal operators (□ (necessity), ◊ (possibility),  (impossibility)) and three deontic operators: O, F, and P. O, F, and P are dyadic sentential operators that take two sentences as arguments and give one sentence as value. Let A and B be two sentences. Then “O[B]A” says that it is obligatory that A given B, “F[B]A” says that it is forbidden that A given B, and “P[B]A” says that it is permitted that A given B. Roughly, according to the semantics “O[B]A” is true in a possible world iff “A” is true in all the best B-worlds, where a B-world is a world in which “B” is true. “F[B]A” is true in a possible world iff “A” is false in all the best B-worlds. And “P[B]A” is true in a possible world iff “A” is true in at least one of the best B-worlds.

The system includes the following definitions: OA (It ought to be the case that A) = _df_ O[⊤]A (where ⊤ is Verum). FA (It is forbidden that A) = _df_ F[⊤]A. PA (It is permitted that A) = _df_ P[⊤]A. A > B (A is better than B) = _df_ P[A ∨ B]⊤ ∧ O[A ∨ B]¬B. A = B (A is as good as B) = _df_ O[A ∨ B]⊥ ∨ (P[A ∨ B]A ∧ P[A ∨ B]B) (where ⊥ is Falsum; O[A ∨ B]⊥ ∨ (P[A ∨ B]A ∧ P[A ∨ B]B) is logically equivalent with P[A ∨ B]⊤ → (P[A ∨ B]A ∧ P[A ∨ B]B)).

When we say that A is as good as B (A is better than B), we can mean many different things. When I am using this expression in this essay, I will usually mean that A is as good as B (A is better than B) *morally* and *all-things considered*. In Sections 3 and 4, I return to the interpretation of some different comparative value expressions.

The definitions above are not obviously true, and in fact, some of them may seem counterintuitive at a first glance. So, let me say a few things in their defence.

1. A definition does not have to be obviously true to be reasonable, fruitful etc. Consider, for instance, the definition of conjunction in terms of negation and implication. A ∧ B can be defined as ¬(A → ¬B). This definition is not obviously true (at least not to everyone). Nevertheless, it is correct and it might be reasonable and fruitful.
2. If we think of the intended semantic interpretation of the system TG, the definitions begin to sound more plausible. Consider, for instance, the definition of better than: A > B (A is better than B) = _df_ P[A ∨ B]⊤ ∧ O[A ∨ B]¬B. In TG, the first conjunct is equivalent to ◊(A ∨ B). So, intuitively “P[A ∨ B]⊤” just asserts that there is at least one possible world in which A ∨ B is true. “O[A ∨ B]¬B” says that in all the best possible worlds in which either A or B is true, not-B is true. In TG, O[A ∨ B]¬B entails O[A ∨ B]A. So, the second conjunct also says that in all the best A ∨ B-worlds, A is true. Suppose A stands for “You give some money to some charity” and B for “You do not give any money to any charity”. Then, according to our definitions, “it is better that you give some money to some charity than if you do not give any money to any charity” is true (in a possible world) iff (i) it is possible that you give some money to some charity or that you do not give any money to any charity and (ii) in all the best possible worlds in which you give some money to some charity or you do not give any money to any charity, it is true that you give some money to some charity and false that you do not give any money to any charity; this seems pretty plausible. Something similar can be said about the other definitions.
3. Even if a definition is not immediately evident or obviously true, it can have reasonable consequences. So, it can be justified or fruitful due to its implications. And in fact, our definitions have many plausible consequences. The definition of “equally good”, for instance, entails that *equally good* is an equivalence relation (i.e. it is reflexive, symmetric and transitive), and the definition of “better than” entails that *better than* is irreflexive, asymmetric and transitive. Whether or not a particular definition is justified, seems to depend upon the plausibility of the whole system in which it occurs. To dismiss a definition solely because it might seem prima facie counterintuitive is surely premature.

Thus, I believe we are justified in considering the consequences of these definitions.

TG is a so-called tableau system. Intuitively, to prove a sentence A in a tableau system, we assume not-A. If this leads to a contradiction (if every branch in the tableau for not-A closes), we conclude that A must be true.^5^ This background information about TG should be sufficient for our purposes in this paper. For more information about TG, see Rönnedal (2009).

Now let us turn to our proof of (P3′). (P3′) contains two parts: (i) and (ii). We begin by proving part (i) and then turn to part (ii).

### Proof of (P3′) (i)

In dyadic deontic logic (P3′) (i) can be symbolised in the following way: (FA ∧ FB) → (¬A = ¬B). This is a theorem in TG. (FA ∧ FB) → (¬A = ¬B) = _df_ (F[⊤]A ∧ F[⊤]B) → (O[¬A ∨ ¬B]⊥ ∨ (P[¬A ∨ ¬B]¬A ∧ P[¬A ∨ ¬B]¬B)). So, to prove part (i) it is enough to establish (F[⊤]A ∧ F[⊤]B) → (O[¬A ∨ ¬B]⊥ ∨ (P[¬A ∨ ¬B]¬A ∧ P[¬A ∨ ¬B]¬B)). I will do this by creating a TG-tableau for the negation of this sentence. Here is the proof.
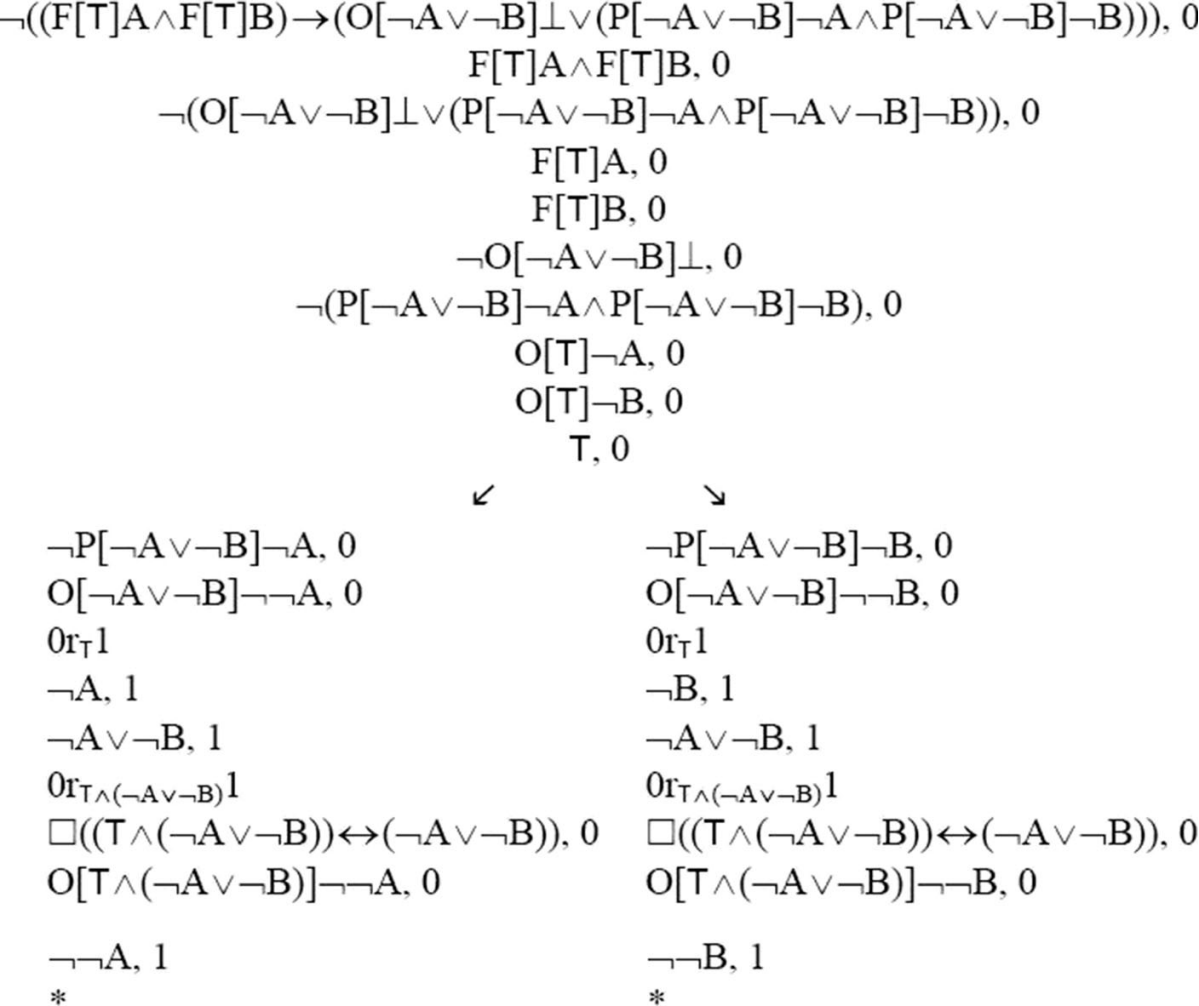


This TG-tableau is closed, since every branch in it is closed. Hence, it constitutes a proof of (FA ∧ FB) → (¬A = ¬B) in TG. Since TG is sound with respect to the class of all so-called H3-models,^6^ (FA ∧ FB) → (¬A = ¬B) is valid in this class of models. In this sense, the principle is valid and cannot be false.

The proof above deserves some comments. The node ⊤, 0 is obtained using the so-called Global Assumption Rule (GA); (GA) says that if A has a tableau proof, then A, i may be added to any open branch in a semantic tableau, for any i. ⊤ is true in every possible world. The step 0r_T_1 on the left (right) branch is derived from the node ⊤, 0, by Tα3.^7^ □((⊤ ∧ (¬A ∨ ¬B)) ↔ (¬A ∨ ¬B)), which occurs on both branches in the tree, is a lemma. It is easy to prove this sentence in TG. Hence, it may be added to any open branch in a tableau by (GA). On the left branch, the step ¬A ∨ ¬B, 1 is obtained from the node immediately above with the help of a derived rule, which we can call “Disjunction Introduction”. According to this derived rule, we may add A ∨ B, i on any open branch in a tableau if we have A, i (or B, i) on this branch. This rule can easily be derived with the help of (GA). Similar remarks apply to the step ¬A ∨ ¬B, 1 on the right branch. The node 0r_T ∧ (¬A ∨ ¬B)_1 on the left branch is obtained from 0r_⊤_1 and ¬A ∨ ¬B, 1 by the rule Tα2.^8^ Similar remarks apply to the node 0r_⊤ ∧ (¬A ∨ ¬B)_1 on the right branch. O[⊤ ∧ (¬A ∨ ¬B)]¬¬A, 0 on the left branch is obtained from O[¬A ∨ ¬B]¬¬A, 0 and □((⊤ ∧ (¬A ∨ ¬B)) ↔ (¬A ∨ ¬B)), 0 by the derived rule DR2. O[⊤ ∧ (¬A ∨ ¬B)]¬¬B, 0 on the right branch is established in a similar way. All other rules that are used in the proof are included in all tableau systems Rönnedal (2009) described.

Now, let us turn to a proof of the second part of (P3′).

### Proof of (P3′) (ii)

(P3′) (ii) can be symbolised in the following way: (PA ∧ PB) → (A = B). This sentence is a theorem in TG. (PA ∧ PB) → (A = B) = _df_ (P[⊤]A ∧ P[⊤]B) → (O[A ∨ B]⊥ ∨ (P[A ∨ B]A ∧ P[A ∨ B]B)). So, if we can establish (P[⊤]A ∧ P[⊤]B) → (O[A ∨ B]⊥ ∨ (P[A ∨ B]A ∧ P[A ∨ B]B)), we have proved part (ii). Accordingly, I will create a TG-tableau for the negation of this sentence. The proof is similar to the proof of part (i).
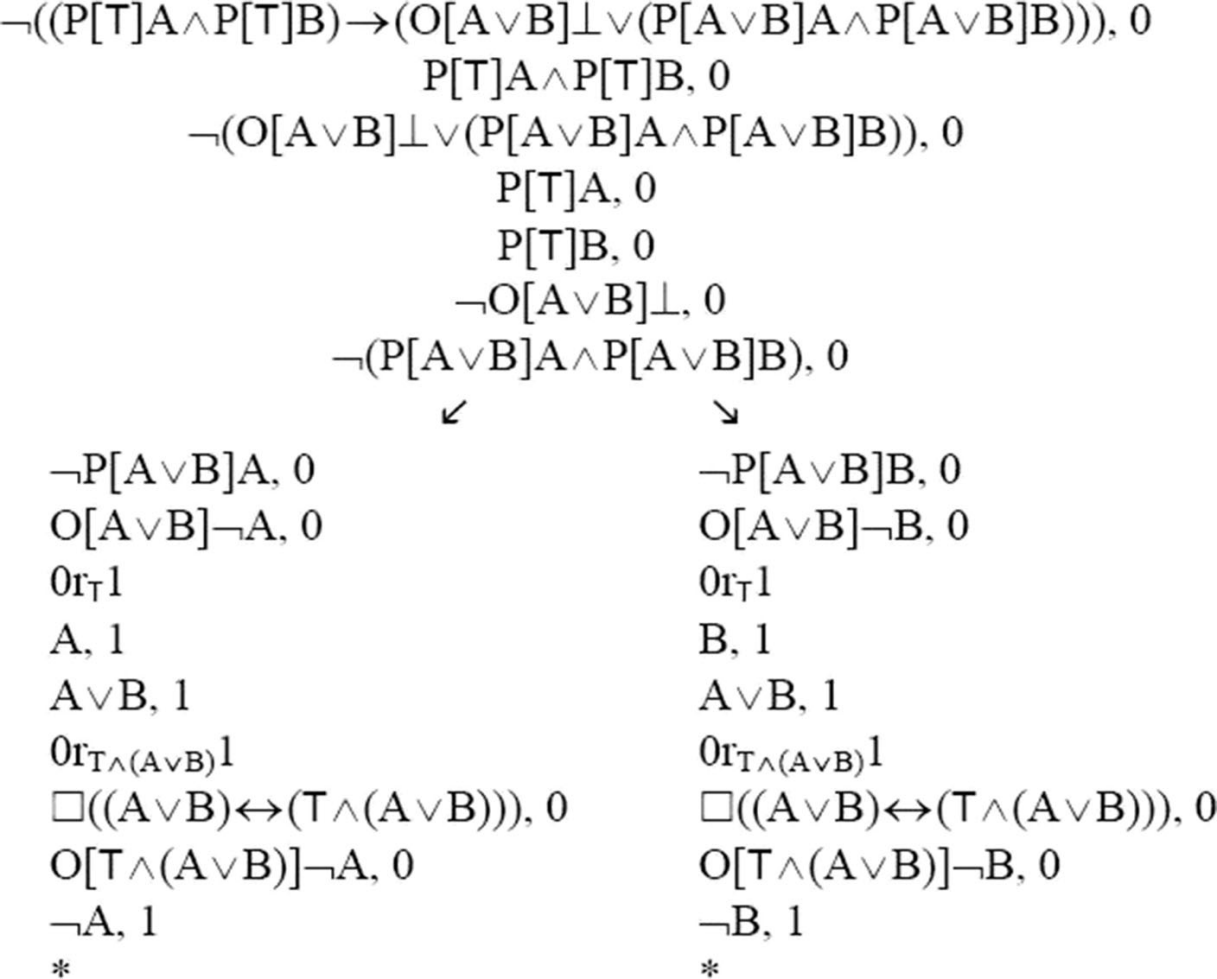


The TG-tableau above is closed, since every branch in it is closed. Hence, it constitutes a proof of (PA ∧ PB) → (A = B) in TG. We observed above that TG is sound with respect to the class of all so-called H3-models (Rönnedal (2009)). Thus (PA ∧ PB) → (A = B) is valid in this class of models. In this sense, part (ii) of (P3′) is also valid and cannot be false.^9^

This concludes our proof of (P3′). It follows that we have also indirectly proven (P3), given that (P3′) is a reasonable interpretation of (P3) and our symbolisation of (P3′) is plausible. The proofs above are obviously correct. Thus, if we want to give up the conclusion, I think we must either reject the definition of “equally good” that we have used in our proofs or abandon the underlying dyadic deontic logic. Therefore, the principle is clearly logically significant.

Now, let us turn to some philosophical arguments against (P3′) and indirectly against (P3).

## Arguments against (P3′) (and indirectly against (P3))

In this section, I will consider two arguments against (P3′) (and hence indirectly against (P3)). The first argument is an argument against part (i) of (P3′) and the second against part (ii) of (P3′).

### An Argument against Part (i) of (P3′)

Consider the following set of sentences:

Set 1

1.1 It is forbidden that you steal an apple.
1.2 It is forbidden that you kill your partner.
1.3 It is not the case that not stealing an apple is as good as not killing your partner.

This set seems to be consistent. Could not all sentences in this set be true simultaneously? But if it is consistent, then (FA ∧ FB) → (¬A = ¬B) cannot be a theorem. If (FA ∧ FB) → (¬A = ¬B) is valid, it is forbidden that you steal an apple and it is forbidden that you kill your partner, then it follows that it is just as good that you do not steal an apple as that you do not kill your partner. However, it is better that you do not kill your partner than if you do not steal an apple. Killing your partner is a much worse crime than is stealing an apple. And if A is better than B, then it is not the case that A is as good as B (this principle is intuitively plausible and also provable in TG). Hence, not stealing an apple and not killing your partner cannot be equally good. Let p = “You steal an apple”, and q = “You kill your partner”. Then the sentences in Set 1 can be symbolised in the following way: (1.1F) Fp, (1.2F) Fq (1.3F) ¬(¬p = ¬q). Obviously, if it is possible that (1.1F)–(1.3F) are true, then (FA ∧ FB) → (¬A = ¬B) cannot be valid.

This argument against (P3′) is similar to some arguments discussed already by Cicero in *Paradoxa Stoicorum*. However, in at least one sense, I think that the problem mentioned above is more problematic for a Stoic than are the examples Cicero mentions. Cicero’s explicit instances of paradox III only concern offenses of *the same sort*. In one of the examples he cites, for instance, he tries to show that all murders are equally wrong.^10^ This might indeed be prima facie counterintuitive, and in Section 4.1 we will see that the principle that if it is forbidden that A and forbidden that B then A is as bad as B is not provable in TG. However, it seems even more difficult to defend the view that all offenses (or the negations of all offenses) of *whatever sort* are equal. Our first counterargument attacks this view.

### An Argument against Part (ii) of (P3′)

Now, consider the following set of sentences:

Set 2

2.1. It is permitted that you keep all your money.
2.2. It is permitted that you give money to some charity.
2.3. It is not the case that keeping all your money is as good as giving money to some charity.

This set also seems to be consistent. But if it is, (PA ∧ PB) → (A = B) cannot be valid. If (PA ∧ PB) → (A = B) is valid, it is permitted that you keep all your money and it is permitted that you give money to some charity, then it follows that it is just as good that you keep all your money as that you give money to some charity. However, it is better that you give money to some charity than it is if you keep all your money. And if this is better, keeping all your money is not as good as giving money to some charity. Let p = “You keep all your money” and let q = “You give money to some charity”. Then the sentences in Set 2 can be symbolised in the following way: (2.1F) Pp, (2.2F) Pq, (2.3F) ¬(p = q). Obviously, given that (PA ∧ PB) → (A = B) is valid, (2.1F)–(2.3F) cannot all be true.^11^

We have now considered two problematic instances of (P3′) and it is easy to come up with countless similar counterintuitive examples. In the light of this, is it not obvious that we should abandon this principle? Is (P3′) not simply a crazy thesis? The arguments against (P3′) in this section certainly seem to be very strong. However, I will now try to defend (P3′) against these counterarguments.

### Some Responses to the Arguments against (P3′)

If we want to defend (P3′), we should try to explain why this principle seems counterintuitive even though it is valid. My hypothesis is that the explanation of this fact, if it is a fact, is that there are several possible interpretations of this thesis that are implausible and invalid even though the principle is valid in our reading. The expressions “forbidden”, “permitted” and “is as good as” can be taken in many incompatible ways.

First of all, we should note that (P3′), in our reading, is not talking about legal norms. When we say that something is forbidden, we do not mean that the law forbids it; and when we say that something is permitted, we do not mean that the law permits it. We mean that something is *morally* forbidden (wrong) *all things considered*, when we say that it is forbidden and we mean that something is *morally* permitted (right) *all things considered* when we say that it is permitted. That it is legally forbidden that A and legally forbidden that B does not mean that not-A and not-B are morally equal. That it is legally permitted that A and legally permitted that B does not entail that A is morally as good as B. It is, for instance, possible that it is legally permitted that you keep all your money and legally permitted that you give money to some charity, even though giving money to some charity is morally better than keeping all your money.

Likewise, when we say that A is as good as B, we do not mean that A and B are *legally* equal. We do not mean that A and B are equally praiseworthy or blameworthy, nor that not-A and not-B are equally praiseworthy or blameworthy. When we say that not stealing an apple is as good as not killing your partner, we do not, for instance, mean that stealing an apple is as blameworthy as killing your partner, nor that stealing an apple ought to be punished as severely as killing your partner. Nor, of course, do we mean that stealing an apple has the same kind of consequences as killing a human being. When we say that A is as good as B, we mean that A is *morally* as good as B *all things considered*. Likewise, when we say that A is better than B, we mean that A is *morally* better than B *all things considered*. Given certain “legal” interpretations of (P3′), (P3′) is implausible and invalid. However, this does not show that (P3′), in our reading, is refuted by the arguments in Section 3.1 and Section 3.2.

It seems that we can use the expressions “A is better than B”, “A is as good as B” etc. in many different senses. According to one interpretation, “A is better than B” means that A is intrinsically better than B and “A is as good as B” means that A is intrinsically as good as B. In other words, “A is better than B” means that A *in itself* is better than B *in itself*, and “A is as good as B” means that A *in itself* is as good as B *in itself*.^12^ In this sense, (P3′) does not appear to be valid. The following claims seem to be possible: “It is forbidden that A and it is forbidden that B, but it is not the case that not-A in itself is as good as not-B in itself”, and “It is permitted that A and it is permitted that B, but A in itself is not as good as B in itself”.

That A in itself is better than B in itself does not imply that A is morally, all things considered, better than B. It can, for instance, be better in itself that you do not go to the dentist than if you go to the dentist because going to the dentist involves some discomfort. However, this does not mean that it all things considered, is better that you do not go to the dentist than if you go to the dentist. All things considered, it is better that you go to the dentist than if you do not go to the dentist, for if you do not go to the dentist, you will get severe toothache and might have to pull out a tooth.

According to a second interpretation, “A is better than B” means that if you must choose between A and B, you should choose A, and “A is as good as B” means that if you have to choose between A and B, it does not matter what you choose. In this sense it clearly seems to be better that you do not kill your partner than if you do not steal an apple. If you are forced to choose between not killing your partner and not stealing an apple, it appears to be the case that you should steal an apple and not kill your partner. And, in fact, this is precisely what is predicted by our dyadic deontic logic. If you must choose between not-A and not-B, if it is not possible that both not-A and not-B, if it is necessary that A or B, then it cannot be that both A and B are forbidden: ¬◊(¬A ∧ ¬B) → ¬(FA ∧ FB) and □(A ∨ B) → ¬(FA ∧ FB) are theorems in TG. So, if it is impossible that you do not steal an apple and that you do not kill your partner, then it is not the case that it is forbidden that you steal an apple and forbidden that you kill your partner. In other words, if it is forbidden that you steal an apple and it is forbidden that you kill your partner, then it is possible not to steal and it is possible not to kill, and it is possible not to steal and not to kill. FA → ◊¬A and (FA ∧ FB) → ◊(¬A ∧ ¬B) are theorems in TG. Conversely, if you are forced to choose between stealing and killing and it is prohibited to kill, then it is not forbidden that you steal an apple. In fact, it follows that you *should* steal. □(A ∨ B) → (¬FA ∨ ¬FB), (□(A ∨ B) ∧ FB) → ¬FA and (□(A ∨ B) ∧ FB) → OA are theorems in TG. Suppose now that it is forbidden that you steal an apple and that it is forbidden that you kill your partner. Then, it follows that it is possible both not to steal an apple and not to kill your partner. Given these assumptions, it is not reasonable to claim that you must choose between not stealing and not killing. In other words, although it is true that you should not kill your partner if you must choose between not stealing and not killing, and that, it in this sense, is better that you do not kill your partner than if you do not steal an apple, it does not follow that it, all things considered, is (morally) better that you do not kill your partner than if you do not steal an apple. If it is forbidden that you steal and it is forbidden that you kill, then it is possible not to steal and not to kill. In all the best possible worlds you neither steal nor kill. Thus, even though it seems that (P3′) is invalid given this interpretation, i.e. if “A is as good as B” means that if you have to choose between A and B it does not matter what you choose etc., it does not follow that our reading of the principle is refuted by the argument in Section 3.1.

In conclusion, when we say that not stealing an apple is as good as not killing your partner, we do not mean that these states of affairs in themselves are equally good, nor that if you had to choose between them, it would not matter what you choose. In most situations, it seems that we can reject (1.3) in Set 1: not stealing an apple is as good as not killing your partner. However, in certain cases, this is not true, for instance if you have to choose between stealing an apple and killing your partner. Then, (1.1) can be rejected. As we have pointed out, if you have to choose between stealing an apple and killing your partner and it is forbidden that you kill your partner, then you should steal an apple. The argument against part (i) of (P3′), therefore, seems to be inconclusive.

Let us say a few more words about the argument against part (ii) of (P3′). Note that {OA, OB, ¬◊(A ∧ B)} and {FA, FB, ¬◊(¬A ∧ ¬B)} are inconsistent, while {PA, PB, ¬◊(A ∧ B)} is consistent. ¬◊(A ∧ B) → ¬P(A ∧ B) is a theorem in TG, but PA ∧ PB does not entail P(A ∧ B). Suppose that you must choose between giving money to some charity and keeping all your money and that it is permitted that you give money to some charity. Even if this is true, it does not follow that it is not permitted that you keep all your money. However, if it is obligatory that you give money to some charity and it is impossible both to give money to some charity and keep all your money, then it is not permitted that you keep all your money.

It seems that the most plausible response to the argument against part (ii) of (P3′) is to reject (2.1), the claim that it is permitted that you keep all your money. If it really is morally better, all things considered, that you give money to some charity than if you keep all your money (and it is permitted that you give money to some charity), it is not all things considered morally permitted that you keep all your money. It follows directly from (P3′) that (¬(A = B) ∧ PB) → ¬PA. (A > B) → ¬(B = A) is both intuitively evident and provable in TG. From this it follows that ((A > B) ∧ PA) → ¬PB, which says that if A is better than B and it is permitted that A, then it is not permitted that B. We can, in fact, prove the following stronger thesis in TG: (A > B) → ¬PB, i.e. if A is better than B, then B is not permitted. This does not entail that it should be legally forbidden not to give money to some charity, nor does it necessarily mean that you should be punished or blamed if you keep all your money. But if it really is better, then it is morally, all things considered, wrong not to give money to some charity. If we do not reject (2.1), we are forced into the conclusion that it is *not* morally better that you give money to some charity than if you keep all your money, and this is highly counterintuitive. If we reject (2.1), however, we seem to end up with a quite demanding ethical system. I return to the question whether it is too demanding or not in section 4.2. In any case, the argument against part (ii) of (P3′) does not seem conclusive.

Let me recapitulate. My suggestion is that we reject (1.3) in Set 1 and (2.1) in Set 2. Set 1 and Set 2 are not really consistent if we understand the sentences in them as we intend in this paper. The fact that they seem consistent can be explained by the hypothesis that the normative and evaluative expressions “forbidden”, “permitted”, and “as good as” can be interpreted in many different ways and that (P3′) is unreasonable given some interpretations. I have mentioned two such interpretations. According to the first, “A is as good as B” says the same thing as “A *in itself* is as good as B *in itself*” etc. According to the second, “A is as good as B” means that if we have to choose between A and B, it does not matter what we choose etc. In our reading, however, the normative and evaluative concepts are interpreted as *all things considered moral concepts*. To my mind, this is the most interesting interpretation of (P3′), at least from a philosophical perspective.

This answer entails that our normative and evaluative expressions are ambiguous, or at least can be used in many senses. However, this seems to be a reasonable conclusion. If the arguments in this section are sound, we do not have to reject (P3′).

## Two Potential Problems

In this section, I will consider two potential problems with the results in this paper. According to the first problem, (P3′) is not a correct interpretation of Cicero’s third paradox, and according to the second problem, it does not matter whether or not (P3′) (or (P3)) is true.

### (P3′) Is not a Correct Interpretation of Cicero’s Third Paradox

One could argue that (P3′) is not a correct interpretation of (P3). According to the first part of (P3), transgressions are equal. I have interpreted this as (P3′) (i). However, (i′), “if it is forbidden that A and it is forbidden that B, then A is as bad as B” is perhaps a better reading of this part of Cicero’s third paradox. It is something like (i′) rather than something like (i) Cicero seems to defend in *Paradoxa Stoicorum*. If this is correct, the following is a better interpretation of (P3):

(P3′′)

(i′) If it is forbidden that A and it is forbidden that B, then A is as bad as B.
(ii) If it is permitted that A and it is permitted that B, then A is as good as B.

Part (i′) of (P3′′) is not a theorem in TG. According to our deontic systems, (P3′′) (i′) might be false. The following counter-model proves this. W = {w_1_, w_2_}; w_2_ > w_1_; A is false in w_2_ and B is false in w_2_, A is true in w_1_ and B false; where W is a set of possible worlds, w_1_ and w_2_ are possible worlds in W, and w_2_ > w_1_ means that w_2_ is better than w_1_. In this model FA, FB and A > B are all true in both w_1_ and w_2_. Thus, according to this model, it is possible that it is forbidden that A and that it is forbidden that B and that A is better than B (for some A and B), and if A is better than B, then it is not the case that A is as bad as B. It follows that we cannot use dyadic deontic logic to prove that this version of the Stoic principle is valid. (P3′′) (ii) = (P3′) (ii). Thus, part (ii) is still provable. However, if (P3′′) is “the” correct interpretation of (P3), it seems that we only can defend the second part of this thesis.

According to dyadic deontic logic, such as the kind used in this paper, it is possible that there are different degrees of wrongdoing, but there are no degrees of right-doing. The following seems to be true. Stealing an apple is not as bad as killing your partner. It is better that you steal an apple than if you kill your partner. This is consistent according to TG. However, not stealing an apple is not better than not killing your partner; not stealing an apple is as good as not killing your partner.

I acknowledge that (P3′′) is probably a better interpretation of Cicero’s reading of (P3) than is (P3′). So why focus on (P3′)? I think that both principles are interesting and that both are prima facie counterintuitive. However, (P3′) is provable in TG while (P3′′) is not. Thus, we can reject (P3′′) without having to abandon either our definitions of “better than” and “equally good” or our underlying dyadic deontic logic. Furthermore, it is not obvious that (P3′′) is a better interpretation of (P3) than is (P3′). Since we do not possess a single complete work by any of the early Stoics, it is very difficult to know for sure what they meant. However, we do know that the Stoics defended (P3) and we can show that (P3′) is valid and that (P3′′) is invalid in dyadic deontic logic. Hence, the so-called principle of charity suggests that (P3′) might be a better interpretation than (P3′′). Of course, this is not a conclusive argument for this reading, and the textual evidence in Cicero (see footnote 2), Sextus Empiricus (*Against the Logicians*, p. 227; 7.422) and Diogenes Laertius (*Lives of Eminent Philosophers*, vol. II, p. 225; 7.120) clearly suggests that (P3′′) is a better interpretation of the Stoic position than is (P3′). If, as seems to be the case, the Stoics argued for (i′) in (P3′′) and not for (i) in (P3′), we might have to conclude that they were wrong about the first part of paradox III: not all transgressions are equal in this sense. At the very least, we cannot use dyadic deontic logic to prove that they were right. For (P3′′) (i′) is invalid in dyadic deontic logic, as we have seen above.^13^ Be that as it may, both (P3′) and (P3′′) share part (ii). Thus, we can still defend this part of (P3); in other words, we can prove the Stoic thesis that right actions are equal. Hence, even if (P3′′) perhaps is a better interpretation of (P3) than is (P3′), the topic of this paper is historically significant.^14^ Furthermore, even if (P3′′) might be historically more interesting than (P3′), (P3′) seems to be at least as interesting as (P3′′) from a philosophical perspective. And from a logical perspective, (P3′) seems to be even more interesting than (P3′′), for we can prove (P3′) in dyadic deontic logic, but we cannot prove (P3′′). Hence, the counter-intuitiveness of (P3′) is more problematic for a deontic logician than the counter-intuitiveness of (P3′′). In conclusion, the choice to focus on (P3′) is not arbitrary.

### It Does not Matter whether or not (P3′) Is True

According to our second problem, it does not matter whether or not (P3′) is true. This principle has no practical consequences. It is perhaps of some minor technical interest to some deontic logicians. But for moral philosophers, let alone for ordinary people, it is an entirely empty and uninteresting thesis. This might seem to be the case, but I will now try to show that (P3′) has important practical consequences.

The main reason why the truth-value of (P3′) matters, or should matter to us, is that (P3′) is connected to the question of how demanding ethics is. In Section 3.3, I argued that the best response to the argument against part (ii) of (P3′) was to reject (2.1). In other words, (P3′) (together with some other very plausible premises) entails the falsity of (2.1). Let us spell out the details of this argument.

1. If it is permitted that you keep all your money and it is permitted that you give money to some charity, then keeping all your money is as good as giving money to some charity. [From (P3′)]
2. It is permitted that you give money to some charity. [Intuitively plausible premise]
3. It is better that you give money to some charity than it is if you keep all your money. [Intuitively plausible premise]
4. If it is better that you give money to some charity than it is if you keep all your money, then keeping all your money is not as good as giving money to some charity. [This follows from the general principle that if A is better than B, then A is not as good as B. This principle is intuitively plausible and can be proven in TG.]

Therefore:

5. It is not permitted that you keep all your money. [From 1 to 4]

This argument is obviously valid and premises (2)–(4) are very plausible. Accordingly, if (P3′) is valid (and hence true) the argument clearly seems to be sound. The conclusion (5) is the negation of (2.1). Q.E.D.

However, this kind of response leads to a quite demanding kind of ethics. If it really is better that you give money to some charity than if you keep all your money (and it is permitted that you give money to some charity), it is not morally permitted that you keep all your money – it is morally wrong. This is a view that coheres well with many kinds of consequentialism that claim that only optimal actions are permitted. And it also fits nicely with a kind of Stoicism that accepts (P3). However, I think it goes against Common Sense morality. Most people probably believe that we, at least sometimes, are morally allowed to perform suboptimal actions. Consider how radical (P3′) is. The principle does not only entail that it is wrong that you keep all your money (if it is better that you give money to some charity than if you keep all your money and it is permitted that you give money to some charity). It is more radical than that. All of the following claims seem to follow. If it is better that you give 10 % of your income to charity than if you give 5 % of your income, then it is not permitted that you give 5 %. If it is better that you give 20 % of your income to charity than if you give 10 %, then it is not permitted that you give 10 %. etc. But where does this stop? Does (P3′) entail that we should give everything we own to charity or perhaps even more than we own? This would surely be a problematic consequence. Our dyadic deontic system has some resources to deal with this problem. We can prove the following sentence in TG: OA → ◊A, which says that something is obligatory only if it is possible. Thus, if it is not possible to give more than we own, it is not obligatory that we do this. At some point, it will probably not be better that we give more and then we can no longer say that it is wrong to give less. But where is the line? How much should we give and how demanding is morality? I will not try to answer these questions in the present paper. I just want to note that if (P3′) is true, morality might be much more demanding than many believe. Therefore, I think it is fair to say that accepting (P3′) can have profound practical consequences for how we ought to live our lives.^15^

## Conclusion

In *Paradoxa Stoicorum*, the Roman philosopher Cicero defends six important Stoic theses. According to one of these principles, paradox III or (P3), (all) transgressions (wrong actions) are equal and (all) right actions are equal. I have investigated one interpretation of (P3) in this paper, namely (P3′). (P3′) consists of two parts: (i) If it is forbidden that A and it is forbidden that B, then not-A is as good as not-B, and (ii) If it is permitted that A and it is permitted that B, then A is as good as B. We have seen that (P3′) can be proven in the dyadic deontic system TG. Therefore, it seems that we have good reasons to accept this principle. However, (P3′) is prima facie counterintuitive. In Section 3, I mentioned a couple of problematic instances of (P3′). If these instances are indeed false, there must be something wrong with my arguments for this thesis. Likewise, if there is something wrong with my arguments for this thesis, it seems that we either have to reject the definitions of “better than”, “equally good” etc. that I use in this paper or abandon the deontic system TG (or both). In Section 3.3, I tried to show that the counterarguments against (P3′) are not conclusive. If the arguments in 3.3 are successful, we do not have to reject (P3′). In Section 4, I addressed the claim that (P3′) is not a correct interpretation of Cicero’s third paradox and the assertion that it does not matter whether (P3′) is true or not. An alternative interpretation of (P3) is (P3′′). (P3′′) consists of two parts: (i′) If it is forbidden that A and it is forbidden that B, then A is as bad as B, and (ii) If it is permitted that A and it is permitted that B, then A is as good as B. (P3′′) is also counterintuitive. However, we cannot prove (i′) in TG. So, if (P3′′) is “the” correct interpretation of (P3), we cannot use dyadic deontic logic of the type we employ in this paper to justify this principle. Since part (ii) is still provable, it seems that at least the second part of paradox III in Cicero’s *Paradoxa Stoicorum* is defensible. In conclusion, the thesis that right actions are equal seems to be defensible if this principle is interpreted as part (ii) in (P3′); the thesis that transgressions are equal seems to be defensible if this principle is interpreted as part (i) in (P3′), but not if it is interpreted as part (i′) in (P3′′). Finally, in Section 4.2, I tried to show why I think (P3′) (and (P3′′)) is practically important and not just theoretically interesting. If (P3′) (or (P3′′)) is indeed true, morality might be much more demanding than many think.

## Footnotes

1 For more information on *Paradoxa Stoicorum*, see Englert (1990), Mehl (2002), Ronnick (1991) and Wallach (1990). For more on Cicero, see Clayton (2016), and Steel (2013). Inwood (2003) is a nice general introduction to Stoicism. See also Baltzly (2013), Brennan (2005), Diogenes Laertius (1972), Long and Sedley (1987), von Arnim (1964). For more on Stoic ethics, see e.g. Annas (2007), Campbell (1985), Cicero (1914), especially Books III and IV, Inwood (1986), Long (1971), Schofield (2003), Sharples (1996), Chapters 5 and 6, Striker (1996), the section on Ethics, White (1990).

2 See e.g. Cicero (1874: 271, 1942: 270–271, 2004: 214–215), Ronnick (1991: 116, 139), Webb (1985: 21, 64). For Cicero’s whole discussion of the paradox, see Cicero (1874: 271–275, 1942: 270–277 (Sect: 20–26), 2004: 214–220), Ronnick (1991: 116–117, 139–140), Webb (1985: 21–25, 64–67).

3 In Cicero (1874, 1942, 2004) the Roman philosopher defends (P3); in Cicero (1914), Book IV, he criticises the principle. Cicero focuses on the first part of the “paradox”. Of course, it should be noted that he considers his discussion as a kind of “rhetorical exercise” that does not necessarily reflect his own beliefs.

4 I want to make two comments about this choice. Firstly, an anonymous reviewer has pointed out that it might be misleading to use “right” and “permitted” interchangeably in the context of Stoicism, since there was a long and intense discussion within Stoicism about whether there were things that were in themselves morally “indifferent”. A Stoic would probably want to make a distinction between what is “right” and what is morally “indifferent”. So it should be noted that “permitted” is not used as synonymous with (morally) “indifferent” in this paper and it is not used as synonymous with (morally) “optional”. As I use the terms, “A is morally optional” is equivalent to “It is permitted that A and it is permitted that not-A”. If we interpret “right” in (P3) as “optional”, we obtain a new possible reading of (ii) in (P3′): (iiK) If it is optional that A and it is optional that B, then A is as good as B. (Some philosophers use “optional” as synonymous with “indifferent”. However, this is not obviously correct. If it is not reasonable to use these words interchangeably, we obtain a new interpretation of (ii): (iiI) If it is indifferent that A and it is indifferent that B, then A is as good as B.) Secondly, some philosophers use “right” as synonymous with “ought” or “obligatory”. If we interpret “right” in this way, we obtain yet another possible reading of (ii): (iiO) If it ought to be the case (is obligatory) that A and it ought to be the case (is obligatory) that B, then A is as good as B. (iiK) and (iiO) are also interesting principles and both are counterintuitive. So it might be interesting to note that (iiK) and (iiO) are provable in dyadic deontic logic. However, I will not investigate these possible interpretations of (P3) in the present paper.

5 For more information on the tableau method, see e.g. Beth (1955, 1959), D’Agostino, Gabbay, Hähnle & Posegga (eds.) (1999), Fitting (1972, 1983, 1999), Jeffrey (1967), Kripke (1959), Priest (2008), Smullyan (1963, 1965, 1966, 1968).

6 In an H3-model, the so-called deontic accessibility relations are defined in terms of a (weak) preference relation between possible worlds. This preference relation is reflexive and transitive. Let A be any sentence and w and w′ be possible worlds. Then w′ is deontically A-accessible from w if and only if w′ is one of the best possible worlds (according to our preference relation) in which A is true. We also assume that if A is true in some possible world, then the set of all the best A-worlds is non-empty. O[B]A is true in w iff A is true in all the possible worlds that are B-accessible from w. P[B]A is true in w iff A is true in at least one possible world that is B-accessible from w, etc. For more on this, see Rönnedal (2009).

7 Tα3 says that if we have A, i on an open branch in a semantic tableau, we may add jr_A_k to this branch, where k is new and A is any sentence. In Rönnedal (2009) it is proved that this rule is sound.

8 Tα2 says that if we have ir_A_j and B, j on an open branch in a semantic tableau, we may add ir_A∧B_j to this branch, where A and B are any sentences. In Rönnedal (2009) it is proved that this rule is sound.

9 Obviously, the proof of (P3′) (ii) is also a proof of (P3′′) (ii) (see Section 4.1) since (P3′′) (ii) = (P3′) (ii).

10 See *Paradoxa Stoicorum*, Sect. 24–25 in the Loeb edition (Cicero (1942)). For references to Cicero’s whole discussion of paradox III, see footnote 2.

11 The argument in Section 3.2 is also a problem for (P3′′) (ii) (see Section 4.1) since (P3′′) (ii) = (P3′) (ii).

12 For more information about the notion of intrinsic value and intrinsic value relations, see Rønnow-Rasmussen and Zimmerman (eds.) (2005).

13 According to Diogenes Laertius (*Lives of Eminent Philosophers*, vol. II, p. 225; 7.121), Heraclides of Tarsus, a disciple of Antipater of Tarsus, and Athenodorus rejected the principle that transgressions are equal. This suggests that the first part of paradox III might have been even more controversial than the second part.

14 I would like to thank an anonymous reviewer for pressing me to be clearer on this point and for making me aware of the reference to Sextus Empiricus (1935).

15 Replace every occurrence of (P3′) in Section 4.2 with an occurrence of (P3′′). Then we obtain an argument that shows that also (P3′′) has important practical consequences for how we ought to live our lives. For it is really part (ii) of (P3′) that is used in step 1 in the argument in Section 4.2, and (P3′) (ii) = (P3′′) (ii). Hence, (P3′′) has exactly the same implications as (P3′) in this case.
